# Phosphorylation of SKAP by GSK3β ensures chromosome segregation by a temporal inhibition of Kif2b activity

**DOI:** 10.1038/srep38791

**Published:** 2016-12-16

**Authors:** Bo Qin, Dan Cao, Huihui Wu, Fei Mo, Hengyi Shao, Jane Chu, Michael Powell, Felix Aikhionbare, Dongmei Wang, Chuanhai Fu, Ping He, Weijun Pan, Wenwen Wang, Xing Liu, Xuebiao Yao

**Affiliations:** 1 Anhui Key Laboratory of Cellular Dynamics and Chemical Biology, University of Science & Technology of China, Hefei 230027, China; 2 Molecular Imaging Center, Atlanta Clinical & Translational Science Institute, Atlanta, GA 30310; 3 Center of Excellence on Molecular Cell Sciences, Chinese Academy of Sciences, Hefei 230026, China; 4 Guangzhou Women and Children’s Medical Center, Guangzhou 510623, China; 5 Shanghai Institutes for Biological Sciences, Chinese Academy of Sciences, Shanghai 200031, China

## Abstract

Chromosome segregation in mitosis is orchestrated by the dynamic interactions between the kinetochore and spindle microtubules. Our recent study shows SKAP is an EB1-dependent, microtubule plus-end tracking protein essential for kinetochore oscillations during mitosis. Here we show that phosphorylation of SKAP by GSK3β regulates Kif2b depolymerase activity by competing Kif2b for microtubule plus-end binding. SKAP is a bona fide substrate of GSK3β *in vitro* and the phosphorylation is essential for an accurate kinetochore-microtubule attachment in cells. The GSK3β-elicited phosphorylation sites were mapped by mass spectrometry and the phosphomimetic mutant of SKAP can rescue the phenotype of chromosome missegregation in SKAP-suppressed cells. Importantly, GSK3β-elicited phosphorylation promotes SKAP binding to Kif2b to regulate its depolymerase activity at the microtubule plus-ends. Based on those findings, we reason that GSK3β-SKAP-Kif2b signaling axis constitutes a dynamic link between spindle microtubule plus-ends and mitotic chromosomes to achieve faithful cell division.

Chromosome dynamics is tightly regulated during the cell cycle. Defects in chromosome segregation gave rise to aneuploidy, which contributed to the pathogenesis and progression of tumor^1^,^2^. A major cause of chromosome mis-segregation is the erroneous attachments between kinetochore (KT) and microtubules (MTs)^3^,^4^,^5^. In addition, microtubule dynamics are key factors for ensuring the proper attachment between kinetochores and MTs. The regulation of microtubule dynamics is associated with a wide range of microtubule-associated proteins (MAPs)^6^,^7^,^8^.

The kinesin-13 family serves as MT depolymerases, which corrects the erroneous KT-MT attachments^9^. In human cells, the kinesin-13 family includes Kif2a, Kif2b, and Kif2c/MCAK. Kif2a localizes to the spindle pole to promote bipolar spindle assembly and to mediate proper chromosome movements during anaphase^10^,^11^. Kif2c/MCAK is currently the best-characterized member among the three proteins. MCAK localizes mainly at centromeres, where it functions to destabilize merotelic MT attachments through MT depolymerase activity^12^,^13^,^14^,^15^. Although the functions of Kif2b are indefinite, Kif2b is evidently required for spindle assembly and chromosome movement^12^,^13^,^14^,^16^,^17^. In consideration of perturbation in Kif2b leads to monopolar or disorganized spindles and lagging chromosomes^18^. Similar to Kif2a and Kif2c/MCAK, Kif2b also exhibits MT depolymerization activity and localizes strongly towards the spindle during metaphase. Therefore, MT depolymerase activity of Kif2b must be down-regulated during metaphase for the preservation of stable attachments between KT and MTs. However, the underlying molecular mechanisms remain undetermined.

Previously, we have shown that SKAP (small kinetochore-associated protein) functions to link dynamic MT plus ends to kinetochores during metaphase by interacting with Mis13, a component of Mis12 complex^19^,^20^. Furthermore, SKAP is also a MAP, distributes at the spindle. SKAP knockdown leads to several mitotic defects, including the formation of multipolar spindle and chromosome misalignment^21^. These mitotic phenotypes are fairly similar to those caused by Kif2b knockdown, thus indicating a potential relationship between SKAP and Kif2b.

The glycogen synthase kinase GSK-3^22^,^23^ functions in several signaling pathways including: glycogen metabolism, Wnt and Hedgehog signal transduction, protein synthesis, mitosis, and apoptosis^24^,^25^,^26^. In human, kinase contains two closely related isoforms, GSK3α and GSK3β^27^,^28^. Interestingly, GSK3β is involved in regulating spindle dynamics and chromosome alignment^29^,^30^. Furthermore, GSK3β is found to accumulate at the centrosomes and localize to the spindle^31^,^32^, while the knockdown of GSK3β results in altered spindle morphology and unnatural chromosomal alignment and mitotic progression^29^,^30^. The results suggest GSK3β may serve as a key mitotic kinase for regulating metaphase spindle dynamics. Indeed, GSK3β can phosphorylate Astrin, a spindle- and kinetochore-associated protein required for proper chromosome alignment in metaphase^33^, and SKAP forms a complex with Astrin. Therefore, GSK3β may also phosphorylate SKAP to spatiotemporally regulate its cellular function.

In present study, we demonstrate Kif2b is a binding protein of SKAP, and the interaction between the two proteins is enhanced during metaphase. Furthermore, GSK3β phosphorylates SKAP to promote its interaction with Kif2b, thus reducing the MT depolymerase activity of Kif2b. The inhibitory effect on Kif2b caused by GSK3β-mediated phosphorylation of SKAP is necessary to maintain proper spindle dynamics and to ensure faithful chromosome segregation.

## Results

### Kif2b is a novel binding protein of SKAP

**Figure 1:**
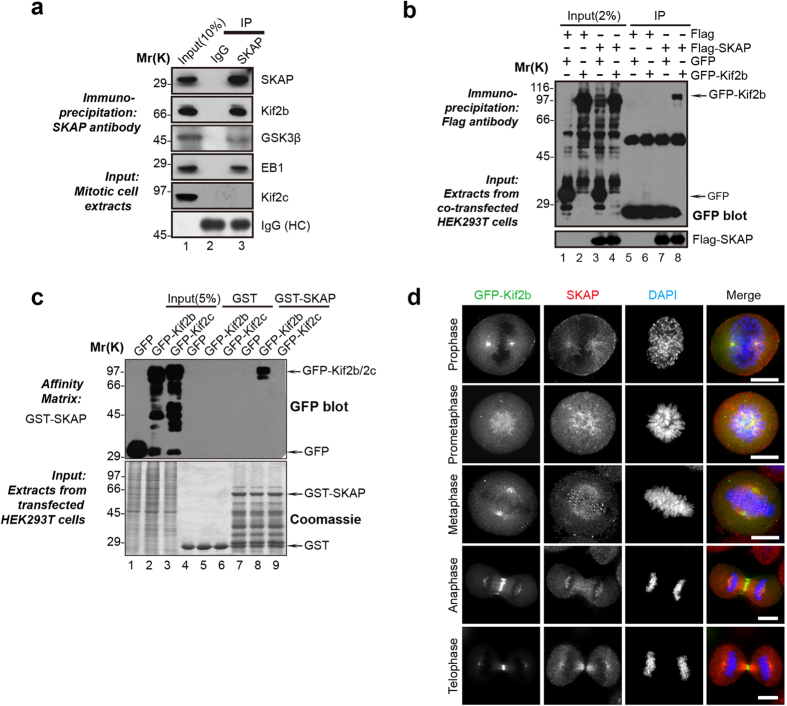
Identification of a novel SKAP-Kif2b interaction in mitosis. (**a**) SKAP complexes were immunoprecipitated from HeLa cell extract with anti-SKAP antibody or control IgG. The isolated complexes were separated using a gel fraction followed by Western blotting with corresponding antibodies. HC, heavy chain. (**b**) Co-immunoprecipitation of exogenous SKAP and Kif2b. Extracts from HEK293T cells, transiently co-transfected with FLAG-SKAP and GFP (lane 3) or GFP-Kif2b (lane 4), were incubated with an anti-FLAG mouse antibody agarose beads. The immunoprecipitates were resolved using SDS-PAGE followed by Western blotting analyses. Upper panel, GFP blot; lower panel, FLAG blot. (**c**) SKAP binds specifically to Kif2b. Purified GST-SKAP proteins were used to isolate GFP-Kif2b or GFP-Kif2c from HEK293T cell lysates. The isolated proteins were fractionated by SDS-PAGE followed by CBB staining (lower) and anti-GFP blotting analysis (upper). (**d**) Representative immunofluorescence images of mitotic cells expressed GFP-Kif2b were fixed and stained with SKAP (red). Scale bars, 10 μm.

Several lines of studies from various laboratories demonstrated the importance of SKAP in mitotic chromosome segregation^19^,^20^. However, it has remained elusive as how SKAP precisely regulates the kinetochore attachment and whether SKAP involves in error correction during mitosis. To further understand these phenotypes caused by SKAP knockdown, we attempted to identify SKAP interacting proteins through affinity purification. To this end, aliquots of HeLa cells synchronized in mitosis were collected followed by generation of clarified cell lysates before absorbed by anti-SKAP affinity matrix and control IgG affinity matrix using the protocol established^20^. As shown in Fig. 1a, Western blotting analysis revealed both Kif2b and GSK3β, but not Kif2c (MCAK), exist in the immunoprecipitates of SKAP. EB1, a known SKAP interacting protein, was also successfully absorbed by SKAP affinity matrix (Fig. 1a). In addition, ectopically expressed SKAP and Kif2b exhibited positive interaction (Fig. 1b, lane 8). Moreover, the recombinant proteins GST-SKAP purified from *E. coli* can specifically pull down GFP-Kif2b, but not GFP-Kif2c, from the HEK293T cell extracts (Fig. 1c). Consequently, these results suggest that SKAP physically interacts with Kif2b.

The finding promoted us to examine the localization of SKAP and Kif2b during mitosis. To visualize the Kif2b, we expressed GFP-tagged Kif2b in HeLa cells, while SKAP was stained with an antibody. As shown in Fig. 1d, both proteins distribute along at the mitotic spindle while SKAP preferentially enriches at the kinetochore during prometaphase-metaphase transition. Thus, we concluded Kif2b is a novel SKAP-binding protein that co-distributes with SKAP at the spindle during mitosis.

### The coiled-coil domain in SKAP is responsible for binding to the C-terminus of Kif2b

**Figure 2:**
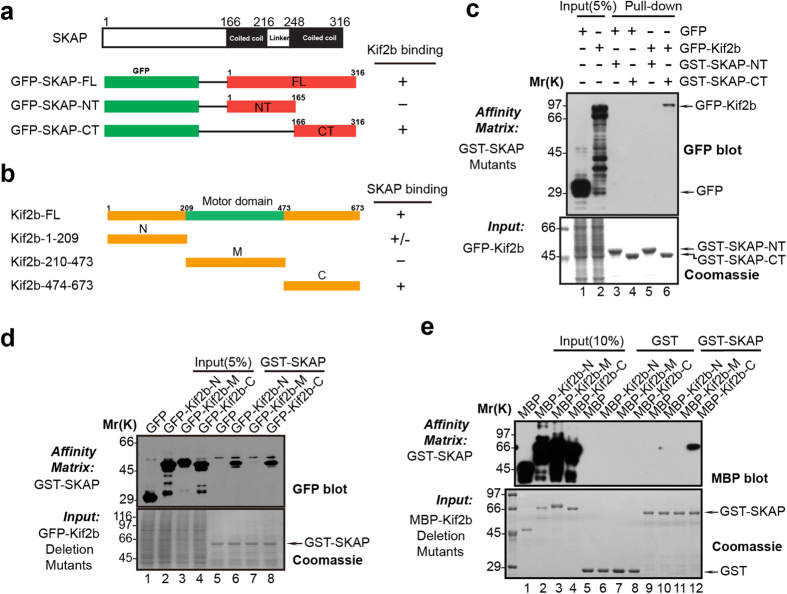
Biochemical characterization reveals a physical link between SKAP C-terminus and Kif2b C-terminus. (**a**,**b**) Schematic drawing of SKAP truncation mutants (**a**) and Kif2b truncation mutants (**b**). +, positive; +/−, weak; −, negative. Residue numbers at domain boundaries are indicated. (**c**) Purified GST-SKAP truncation mutants were used to absorb GFP-Kif2b truncation mutants from HEK293T cell lysates. Western blotting analysis, with anti-GFP antibody, indicated the specific association. (**d**) SKAP binds to Kif2b N (amino)-terminus as well as C (carboxyl)-terminus. Purified GST-SKAP proteins were used to isolate GFP-Kif2b deletion mutants from HEK293T cell lysates, and the isolated proteins were fractionated by SDS-PAGE followed by CBB staining (lower) and an anti-GFP blotting analysis (upper). (**e**) SKAP binds directly to Kif2b. Using purified GST-SKAP proteins as matrices, a GST pull-down assay was performed to bind purified MBP-tagged Kif2b deletion mutants. Western blot (upper panel) with anti-MBP antibody demonstrated a specific interaction.

Next, we aimed to map the interface between SKAP and Kif2b physical contact. Based on the characteristic domain structures we created a series of deletion truncation mutants for SKAP and Kif2b, respectively (Fig. 2a and b). To pinpoint the region of SKAP binding to Kif2b, we performed GST pull-down assays using *E. coli*-produced recombinant GST-tagged SKAP full length as well as its deletion truncation mutants, and HEK293T cell extracts containing ectopically expressed GFP-Kif2b. As shown in Fig. 2c, Western blotting analysis displayed the C-terminus of SKAP binding to Kif2b (lane 6). Similarly, the N- and C- terminal regions outside of the Kif2b motor domain were important for interacting with SKAP (Fig. 2d and e), with the C-terminus displaying a stronger interaction. Therefore, we concluded that the C-terminus of SKAP and Kif2b are responsible for the interaction of these two proteins.

### SKAP inhibits the MT depolymerase activity of Kif2b

**Figure 3:**
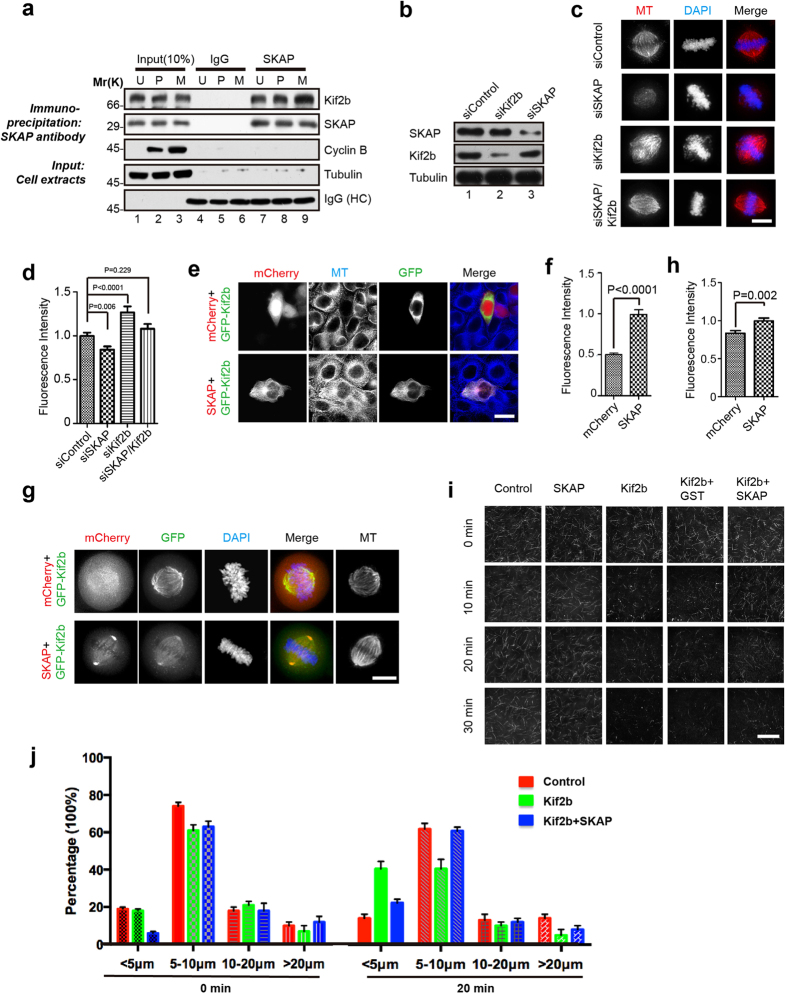
SKAP inhibits depolymerase activity of Kif2b to ensure spindle microtubule stability in metaphase. (**a**) Co-immunoprecipitation of SKAP with endogenous Kif2b. HeLa cells were either unsynchronized (U) or synchronized in prometaphase (P) or metaphase (M). The lysates were subjected to immunoprecipitation with IgG or SKAP antibody. The precipitates and cell lysates were analyzed using immunoblot with antibodies against SKAP, Kif2b, cyclin B and tubulin. (**b**) Knockdown efficiency of SKAP and Kif2b siRNAs. (**c**) Representative immunofluorescence images of HeLa cells co-transfected with indicated siRNAs and GFP. 72 h post transfection, HeLa cells were treated with MG132, then fixed and stained with MTs (red) and DNA (blue). Scale bars, 10 μm. (**d**) Relative fluorescence intensity of bipolar spindle to DNA in (**c**) were calculated and graphed. Bars indicate means ± SEM from three independent experiments. Statistical significance was evaluated through the Student’s *t*-test. (**e**) Representative immunofluorescence images of HeLa cells co-expressing GFP-Kif2b with mCherry or mCherry-SKAP. Cells were fixed and stained with MTs (blue). Scale bars, 10 μm. (**f**) The ratios of the MT fluorescence in transfected cells to that of untransfected cells within the same field of view in *e* were calculated and graphed. Bars indicate means ± SEM from three independent experiments. Statistical significance was evaluated by the Student’s *t*-test. (**g**) Representative immunofluorescence images of HeLa cells co-expressing mCherry or mCherry-SKAP and GFP-Kif2b. 36 h post transfection, HeLa cells were treated with MG132, then fixed and stained with MTs and DNA (blue). Scale bars, 10 μm. (**h**) Relative fluorescence intensity of bipolar spindle to DNA in *g* were calculated and graphed. Bars indicate means ± SEM from three independent experiments. Statistical significance was evaluated by Student’s *t*-test. (**i**) Representative images of GMPCPP-stabilized rhodamine-labeled MTs incubated with indicated proteins for corresponding incubation time. FLAG-Kif2b was mixed with GST or GST-SKAP and incubated with GMPCPP-stabilized rhodamine-labeled MTs. Images were taken at corresponding incubation time at 25 °C. MTs were then examined by TIRF microscope at the indicated time. Scale bars, 20 μm. (**j**) Statistical analysis of the microtubule length at 0 min and 20 min as in (**i**).

We next analyzed the interaction between SKAP and Kif2b in a cell cycle dependent manner, and found that SKAP exhibited a stronger binding to Kif2b in metaphase cell lysates than those of in interphase and prometaphase as shown by immunoprecipitation with cells synchronized at the respective cell-cycle phases (Fig. 3a). To understand the biological significance of the interaction of SKAP with Kif2b, we sought to investigate the effects of SKAP or Kif2b absence on spindle MT organization. As shown in Fig. 3b, transfection of SKAP siRNA and Kif2b siRNA efficiently repressed the expression of corresponding proteins after 48 h. In addition, measurements of the fluorescence intensity of spindle MTs versus DNA showed SKAP knockdown caused a significant reduction of spindle MT (Fig. 3c), which suggests the role of SKAP in spindle MT stabilization. The findings are consistent with our previous results^34^. The total MT populations in cells transfected with Kif2b siRNA were greater than those in control cells, as expected^17^. Interestingly, double suppression of SKAP and Kif2b had no significant effect on the spindle MT intensity (Fig. 3d). These results suggest SKAP and Kif2b antagonize with each other to regulate spindle MT dynamics.

Since Kif2b is a MT depolymerase, we reasoned SKAP may stabilize MTs by inhibiting the MT depolymerase activity of Kif2b. To test this hypothesis, GFP-Kif2b and mCherry-SKAP (or mCherry) were co-expressed in HeLa cells, and then the MT density was analyzed by measuring the microtubule fluorescent intensity. As shown in Fig. 3e–h, the MT density in GFP-Kif2b and mCherry-SKAP co-expressing cells was similar to the MT density in untransfected cells; however, the MT density was significantly reduced in GFP-Kif2b and mCherry co-expressing cells. The results were further confirmed by our *in vitro* MT reconstitution assays, since the presence of SKAP significantly inhibited the MT depolymerization activity of Kif2b (Fig. 3i and j and Supplemental Fig. S1). Collectively, these results suggest SKAP functions as an inhibitory factor of Kif2b in regulating MT dynamics.

### SKAP is a novel substrate of GSK3β

**Figure 4:**
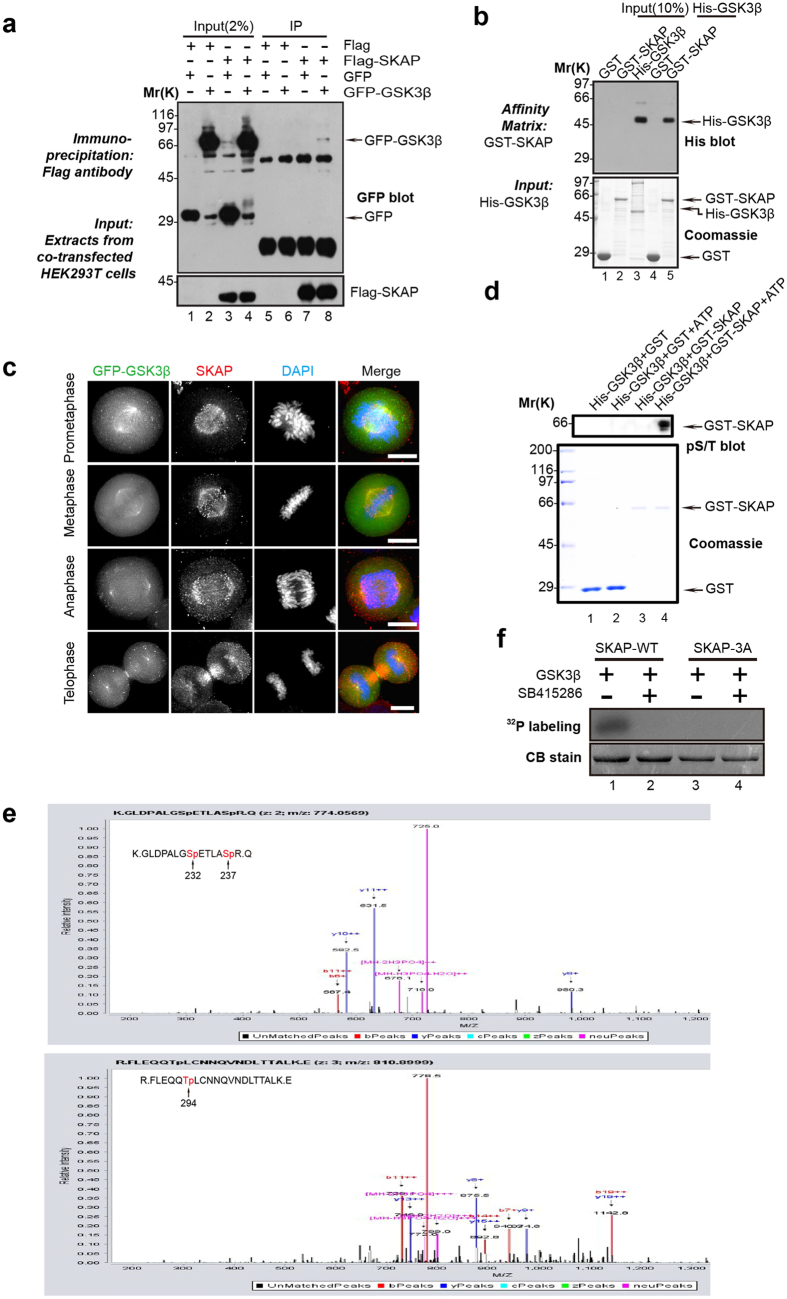
SKAP is phosphorylated by GSK3β. (**a**) Co-immunoprecipitation of exogenous SKAP and GSK3β. Extracts from HEK293T cells were transiently co-transfected with both FLAG-SKAP and GFP (lane 3) or GFP-GSK3β (lane 4). The extracts were incubated with an anti-FLAG mouse antibody agarose beads. The immunoprecipitates were resolved by SDS-PAGE followed by Western blotting analyses. Upper panel, GFP blot; lower panel, FLAG blot. (**b**) SKAP directly binds to kinase GSK3β. Using purified GST-SKAP truncation mutants as the matrices, a GST pull-down assay was performed to bind purified His-tagged GSK3β. Western blotting with anti-His antibody showed a specific interaction (lane 5). (**c**) Representative immunofluorescence images of mitotic cells expressing GFP-GSK3β were fixed and stained with SKAP (red). Scale bars, 10 μm. (**d**) Bacterially recombinant GST-SKAP and GST were incubated with GSK3β kinase purified from Sf9 cells in an *in vitro* phosphorylation reaction. Samples were separated by SDS-PAGE followed by CBB staining and Western blot with an anti-p-ser/thr antibody. (**e**) Identification of sites on SKAP phosphorylated by GSK3β. Recombinant GST-SKAP was phosphorylated by GSK3β *in vitro* in the presence of ATP as described in “Materials and Methods”, while the phosphorylation sites on SKAP were identified through Mass Spectrometry. The three phosphorylation sites (Ser232, Ser237, Thr294) identified were shown as red. (**f**) Recombinant GST-SKAP-WT and its mutant GST-SKAP-3A were incubated with GSK3β in the presence or absence of GSK3β inhibitor SB415286 as described in “Materials and Methods”.

In our SKAP affinity purification, the protein kinase GSK3β was also identified as a binding protein of SKAP (Fig. 1a). We then asked whether GSK3β could phosphorylate SKAP and subsequently regulate its function. First, we confirmed the interaction between GSK3β and SKAP through co-immunoprecipitation assays using cells expressing FLAG-SKAP and GFP-GSK3β (Fig. 4a) and GST pull-down assays using recombinant proteins GST-SKAP and His-GSK3β (Fig. 4b). Both of the results suggested a physical link between the kinase GSK3β and SKAP. Furthermore, immunofluorescent co-staining analysis displayed SKAP and GSK3β colocalized at the spindle (Fig. 4c).

The interaction between SKAP and GSK3β raised the possibility that SKAP might be a novel substrate of GSK3β. To test this idea, *in vitro* phosphorylation assays were performed using recombinant proteins, GST-SKAP and His-GSK3β, in the presence of γ-^32^P-ATP (Supplemental Fig. S2). As shown in Fig. 4d, GST-SKAP, but not GST, was phosphorylated by GSK3β detected by immunoblot with an anti-p-Ser/Thr antibody. To pinpoint the phosphorylation sites, we removed the GST-SKAP protein band for mass spectrometric analyses^15^. As annotated in Fig. 4e, three phosphorylation sites were identified at the C-terminal region of SKAP (Ser232, Ser237 and Thr294). To validate the three sites are indeed phosphorylated by GSK3β, we performed *in vitro* phosphorylation assays using wild-type and non-phosphorylatable SKAP mutant, in which the three sites were replaced by alanine (designated as SKAP-3A). As shown in Fig. 4f (upper panel), incubation of γ-^32^P-ATP with various recombinant SKAP proteins in the presence of GSK3β kinase resulted in an efficient incorporation of ^32^P into the recombinant GST-SKAP wild-type, but not non-phosphorylatable GST-SKAP-3A, protein (Fig. 4f, lane 3). Furthermore, SKAP phosphorylation was eliminated upon the addition of the GSK3β kinase inhibitor SB415286 (30 μM) (Fig. 4f, lane 2). Thus, we concluded GSK3β phosphorylates SKAP at Ser232, Ser237 and Thr294.

### Phosphorylation of SKAP by GSK3β inhibits Kif2b depolymerase activity

**Figure 5:**
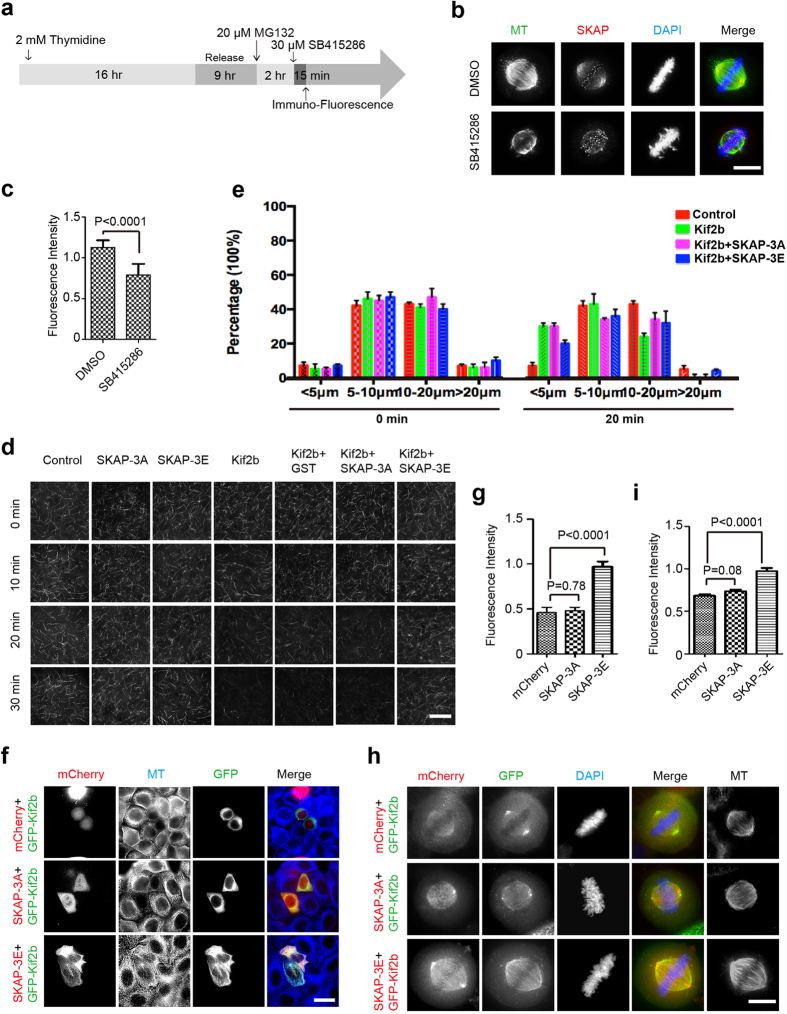
Phosphorylation of SKAP inhibits Kif2b depolymerase activity. (**a**) Schematic overview of the experimental procedure of drug treatment for immunofluorescence (see Materials and Methods). (**b**) Representative immunofluorescence images of spindle MTs exhibit higher fluorescence intensity in control cells than that in SB415286-treated cells. Scale bars, 10 μm. (**c**) Relative fluorescence intensity of bipolar spindle to DNA in (**b**) were calculated and graphed. Bars indicate means ± SEM from three independent experiments. Statistical significance was evaluated using Student’s *t*-test. (**d**) Representative images of GMPCPP-stabilized rhodamine-labeled MTs incubated with indicated protein at corresponding time. Purified FLAG-Kif2b was mixed with GST-SKAP-3A or GST-SKAP-3E and then incubated with GMPCPP-stabilized rhodamine-labeled MTs. Images were taken at corresponding incubation time at 25 °C. MTs were then examined by TIRF microscope at indicated time. Scale bars, 20 μm. (**e**) Statistical analysis of the microtubule length at 0 min and 20 min in (**d**). (**f**,**h**) Representative immunofluorescence images of HeLa cells co-expressing GFP-Kif2b and mCherry-SKAP-3A or mCherry-SKAP-3E in interphase (**f**) and metaphase (**h**). Cells were fixed and stained with MTs (blue). Scale bars, 10 μm. (**g**) The ratios of MT fluorescence in transfected cells to that of untransfected cells, within the same field of view in (**f**), which were calculated and graphed. Bars indicate means ± SEM from three independent experiments. Statistical significance was evaluated by Student’s *t*-test. (**i**) Relative fluorescence intensity of bipolar spindle to DNA in (**h**) were calculated and graphed. Bars indicate means ± SEM from three independent experiments. Statistical significance was evaluated by unpaired Student’s *t*-test.

Previous studies have reported GSK3β, which predominantly localizes at spindle MTs in metaphase, plays an important role in controlling the microtubule dynamics during mitosis^29^. However, the underlying molecular mechanisms remain elusive. First, we tested whether the inhibition of GSK3β kinase activity could destabilize spindle MTs in metaphase. HeLa cells were arrested in metaphase before the addition of GSK3β inhibitor SB415286 (Fig. 5a). As shown in Fig. 5b and c, the fluorescence intensity of spindle MTs was significantly decreased in the cells treated with SB415286, indicating that the kinase activity of GSK3β is required for maintaining spindle MT stability during metaphase.

The results led us to hypothesize GSK3β may regulate spindle MT stability through modulating the activity of SKAP and Kif2b. To test this hypothesis, we carried out MT depolymerization assays *in vitro*. FLAG-Kif2b was briefly purified from HEK293T cells, while GST-SKAP mutants were purified from *E.coli*. The purified proteins were incubated with rhodamine-labelled GMPCPP-stabilized MTs for indicated time. As shown in Fig. 5d and e, SKAP mutants, unlike Kif2b, did not cause MT depolymerization. However, SKAP-3E, but not SKAP-3A, significantly inhibited the MT depolymerization activity of Kif2b. The same results were found with coexpression of SKAP-3E and Kif2b, but not the coexpression of SKAP-3A and Kif2b (Fig. 5f–i). Taken together, we concluded GSK3β-mediated phosphorylation of SKAP inhibits the MT depolymerase activity of Kif2b, which governs the spindle stability during metaphase.

### GSK3β-mediated phosphorylation promotes SKAP binding affinity to Kif2b

**Figure 6:**
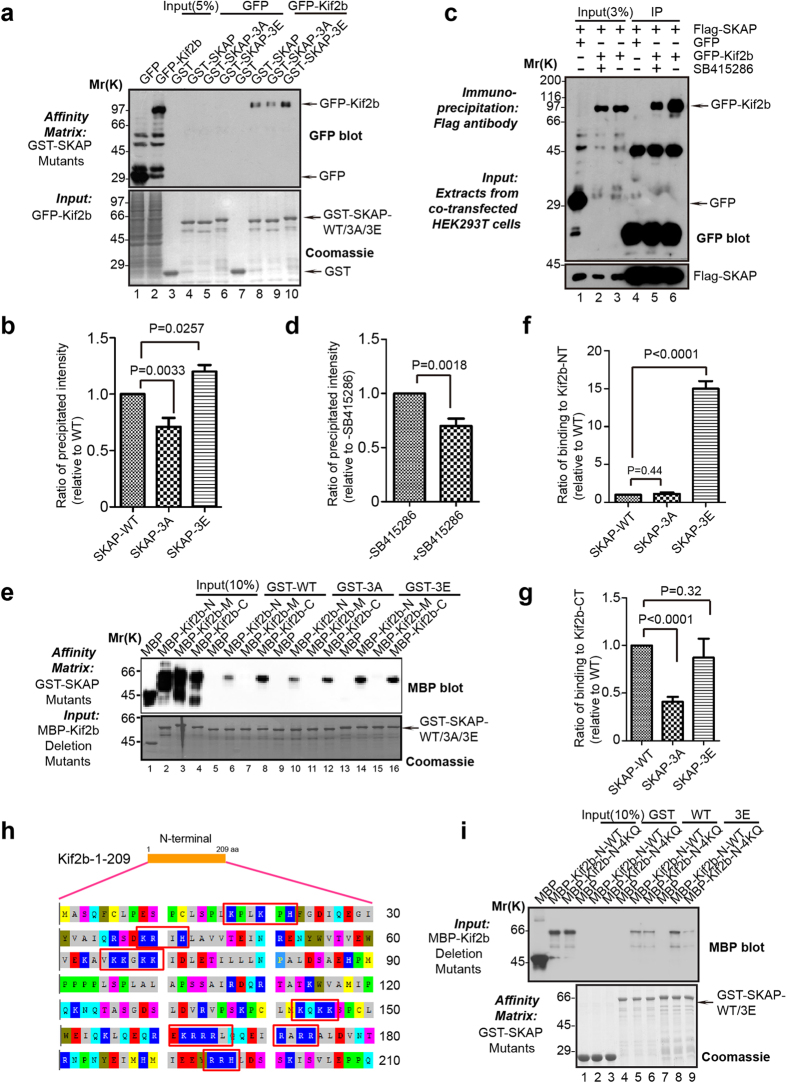
Phosphorylation of SKAP increases its binding affinity to Kif2b. (**a**) Purified GST-SKAP and SKAP mutants were used as matrices to isolate GFP-Kif2b from HEK293T cell lysates. The isolated proteins were then fractionated by SDS-PAGE followed by CBB staining (lower) and anti-GFP blot (upper). (**b**) Statistical analysis of binding efficiency in (**a**). The ordinate indicates the relative binding ratio of GFP-Kif2b to GST-SKAP and its mutants; the abscissa indicates the corresponding binding assay shown in (**a**). (**c**) Co-immunoprecipitation of exogenous SKAP and Kif2b. 36 h after transfection with FLAG-SKAP and GFP-Kif2b, the HEK293T cells were treated with SB415286 for 30 min (lane 5). The cell extracts were incubated with an anti-FLAG antibody agarose beads, and the immunoprecipitates were resolved by SDS-PAGE followed by Western blots. (**d**) Statistical analysis of binding activity in (**c**). The ordinate indicates the binding ratio of GFP-Kif2b and FLAG-SKAP; the abscissa indicates the corresponding binding assay shown in (**c**). (**e**) Purified GST-SKAP and its mutants were used as matrices to isolate purified MBP-Kif2b deletions. The isolated proteins were fractionated by SDS-PAGE followed by CBB staining (lower) and anti-MBP blot (upper). (**f**–**g**) Statistical analysis of binding ratio in (**e**). The ordinate indicates the binding ratio of MBP-Kif2b-NT (**f**) or MBP-Kif2b-CT (**g**) to GST-SKAP wild-type and its mutants, while the abscissa indicates the corresponding binding assay shown in (**e**). Note that there is no significant difference between wild-type SKAP and phospho-mimicking SKAP-3E (*p* = 0.32) bound to Kif2b-C while binding efficiency of non-phosphorylatable SKAP-3A to Kif2b-C was significantly reduced (*p* < 0.001; n = 3). (**h**) The primary sequence of Kif2b N-terminal truncation. The red boxes indicate the selected basic amino acid groups. (**i**) Purified GST-SKAP wild-type and its mutants were used as matrices to isolate purified MBP-Kif2b-N and its mutants, then they were fractionated by SDS-PAGE followed by CBB staining (lower) and anti-MBP blot (upper). 4KQ means K66, K67, K69 and K70 have been mutated to Q.

To address how GSK3β-mediated phosphorylation of SKAP acts to inhibit the MT depolymerase of Kif2b, we sought to test whether the phosphorylation could affect the interaction between SKAP and Kif2b. Initially, we performed a GST pull-down assay. As shown in Fig. 6a, Western blotting analysis exhibited different amounts of GFP-Kif2b succumbing to the pull-down by GST-SKAP, and the phospho-mimetic mutant SKAP-3E displayed the strongest interaction (Fig. 6a and b). The same results were found when coimmunoprecipitation experiments were performed using FLAG-SKAP and GFP-Kif2b expressing cells. As shown in Fig. 6c and d, the interaction between SKAP and Kif2b was weakened upon addition of SB415286, the GSK3β inhibitor. Careful examination of the N-terminal amino acid sequences of Kif2b showed this region is rich with positively charged residues (Lys & Arg), indicating that the electrostatic force may be involved in the regulation of the interaction between SKAP and Kif2b. Evidently, GST pull-down assays demonstrated SKAP-3E interacting more strongly with Kif2b N-terminus than other SKAP variants (WT & 3A) (Fig. 6e–g), while mutations of the four basic residues (K66, K67, K69, K70) within the N-terminal of Kif2b to Glutamine significantly blocked its interaction with SKAP-3E (Fig. 6h and i). Hence, we conclude GSK3β-mediated phosphorylation of SKAP enhances the interaction between SKAP and Kif2b, and the enhanced binding may further inhibit the MT depolymerase activity of Kif2b.

**Figure 7:**
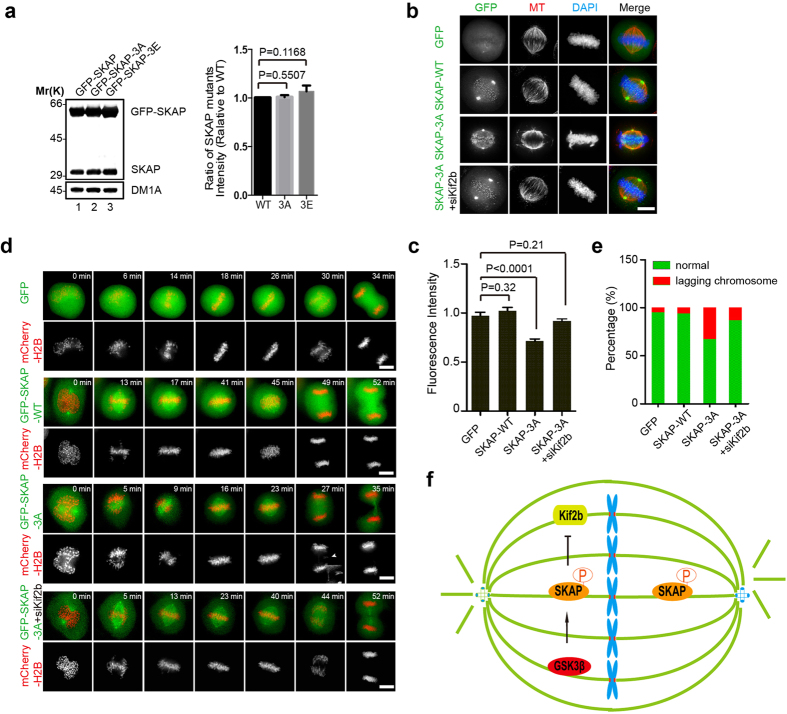
Phosphorylation of SKAP mediated by GSK3β ensures chromosome segregation in mitosis. (**a**) Immunoblot of HeLa cell lysates expressing GFP-SKAP-WT, GFP-SKAP-3A or GFP-SKAP-3E, with anti-SKAP antibodies. Quantification is shown on the right. Bars represent means ± SD, n = 3. (**b**) Representative immunofluorescence images of HeLa cells overexpressing GFP-SKAP or its mutants and indicated siRNA. 72 h post transfection, HeLa cells were arrested with MG132, and then fixed and stained with MTs and DNA (blue). Scale bars, 10 μm. (**c**) Relative fluorescence intensity of bipolar spindle to DNA shown in “**b**” was calculated and graphed. Bars indicate means ± SEM from three independent experiments. Statistical significance was evaluated by Student’s *t*-test. (**d**) Real-time imaging of chromosome movement in cells overexpressing GFP-SKAP or its mutants with the indicated siRNA. Cells were co-transfected with mCherry-H2B. 72 h post transfection, cells were observed with a DeltaVision system. Scale bars, 10 μm. (**e**) Statistical analysis of lagging chromosome in *d*. At least 30 cells were analyzed from three independent experiments for each construct. (**f**) Proposed model of GSK3β-SKAP-Kif2b axis in maintaining metaphase spindle stability.

To explore the functional significance concerning the inhibition of Kif2b by GSK3β-mediated phosphorylation of SKAP, we examined the metaphase chromosome alignment in HeLa cells expressing GFP-SKAP and its phosphorylation mutants. HeLa cells were transfected with GFP-SKAP-WT, GFP-SKAP-3A or GFP-SKAP-3E, and synchronized to metaphase via MG132. As expected, the relative spindle MT density in GFP-SKAP-3A-transfected cells was lower than that in GFP-SKAP-WT or GFP-SKAP-3E transfected cells (Fig. 7a), which was possibly caused by a greatly reduced MT depolymerase activity of Kif2b. In support of this notion, suppression of Kif2b restored the relative spindle MT density in SKAP-3A expressing cells (Fig. 7b and c). Therefore, we concluded metaphase spindle stability required the inhibition of Kif2b activity.

To assess the effects of SKAP-WT and the phosphorylation mutants on mitotic progression, time-lapse imaging experiments were performed. HeLa cells transfected with GFP-SKAP-WT, GFP-SKAP-3A or GFP-SKAP-3E were synchronized, and then time-lapse imaging was conducted at 72 h post-transfection. As shown in Fig. 7d and e, over-expression of GFP-SKAP-3A resulted in chromosome instability, which is evident by the dramatic increase of cells exhibiting lagging chromosomes during anaphase. We also examined the cell phenotype when Kif2b was suppressed in SKAP-3A expressing cells. The results revealed that knockdown of Kif2b rescued chromosome instability resulted from SKAP-3A expression in cells (Fig. 7d and e). Thus, we conclude that cell-cycle dependent regulation of Kif2b activity by SKAP is critical for faithful mitotic progression.

## Discussion

Cancer cells often display aneuploidy, a consequence of chromosome mis-segregation likely caused by aberrant KT-MT attachments^35^. Generally, a wide range of MAPs are involved in regulating spindle microtubule dynamics to ensure proper chromosome segregation. However, how these MAPs work in concert to orchestrate microtubule and spindle dynamics is unclear. In this study, we demonstrated SKAP physically interacts with Kif2b to regulate MT dynamics and maintain the integrity of the spindle (Fig. 7f).

MT dynamics^36^ is mediated by highly coordinated polymerization and depolymerization of MTs, which ensures the integrity of chromosome segregation^37^. Kinesin-13 family is widely known as a key factor for depolymerizing microtubule ends^38^,^39^. Intriguingly, our results revealed SKAP interacts with Kif2b both *in vitro* and *in vivo* but not with Kif2c. In addition, Kif2b co-localized with SKAP through spindle MTs during mitosis. Previous studies have shown that SKAP directly interacts with Mis13 through its CC2 domain to localize at kinetochores^19^. We have demonstrated the CT of SKAP also binds to Kif2b to inhibit depolymerase activity. Consequently, we conclude SKAP may interact with different proteins through its CT in pursuance of playing roles in different cellular processes. It would be remarkably important to delineate the structural basis and modifications control under the interactions of SKAP with different proteins.

In our study, we reported SKAP can be phosphorylated through GSK3β, and three phosphorylation sites have been identified in the C-terminal domain of SKAP. The SKAP-Kif2b interaction is strengthened by the phosphorylation. The phosphomimetic SKAP mutant, SKAP-3E, inhibits the depolymerization of spindle MTs, which are caused by depolymerase activity of Kif2b. Cells expressed with non-phosphorylatable SKAP mutant SKAP-3A exhibit destabilized spindle MTs and lagging chromosome, which suggests the importance of SKAP phosphorylation by GSK3β. Phosphorylation by GSK3β regulates the stability and integrity of spindle MTs, as well as accurate chromosome segregation. Derived from our results, phosphorylation of SKAP by GSK3β increases the negative charge in SKAP C-terminus. The result is an enhanced binding ability of SKAP to Kif2b N-terminus, which possesses several basic amino acid groups. In general, GSK3β exhibits a preference for pre-phosphorylated (primed) substrates, recognizing the consensus sequence S/T-X-X-X-Phospho-S/T. However, recent studies show that a large collection of GSK3β substrates does not need priming phosphorylation, indicating that GSK3β phosphorylates its substrates using two distinctly different mechanisms. We have examined the three phosphorylation sites on SKAP which do not require pre-phosphorylation, demonstrating that GSK3β phosphorylates SKAP without requirement of pre-phosphorylation and is independent of canonical pathway.

Kif2c, the most well-known member of kinesin-13 family^40^, was reported to switch its conformation when phosphorylated, which altered its MT binding affinity and MT depolymerization activity^15^. Aurora B-mediated phosphorylation converts Kif2c from a “closed” to an “open” conformation, resulting in Kif2c disassociation from MT^41^,^42^. In addition, Kif2c also cooperates with TIP150 to promote MT dynamics and modulate the mechanical rigidity of the cells during entosis by Aurora B-mediated phosphorylation^43^. Based on our previous results, Kif2b may have a similar conformational change, which is associated with the regulation of depolymerization activity. We also presume Kif2b exhibits a higher depolymerization activity in a “closed” conformation, while the active form of Kif2b might be “closed” rather than “open”. The binding of SKAP to Kif2b N-terminal may disrupt the “closed” conformation, thereby converting Kif2b from a “closed” to “open” conformation. The atomic resolution of Kif2b structure will provide us with more comprehensive data about the regulation of its depolymerization activity. Moreover, an important future experiment would be taking advantage of FRET reporters to visualize the conformational dynamics of Kif2b in space and time of live cell division. These dynamic changes are closely associated with regulated activity, whereas the conformational change in Kif2c was induced by Aurora B phosphorylation. It is also noteworthy to study the potential modification on Kif2b, which might lead to conformational change of Kif2b.

As previously reported, SKAP localizes at spindle poles, spindle microtubules and kinetochores in metaphase^44^. There are two pools for SKAP, one for proper spindle microtubule growth and another for proper MT-KT attachment. Previous study revealed the Astrin-SKAP complex serves as a unique class bound MAPs, which localizes to the kinetochores following microtubule-end association. Additionally, the complex remains at the KT-MT interface to maintain the stability of MTs. Combined with the inhibiting effect action of SKAP on depolymerase activity of Kif2b, we speculate the pool of SKAP on spindle MTs might stabilize microtubules through inhibiting depolymerase activity of Kif2b. SKAP also cooperates with mitotic kinesin CENP-E to govern dynamic KT-MT interaction for faithful chromosome segregation^20^, which promotes the speculation of CENP-E’s role in translocating SKAP from spindle to kinetochore. Our previous study discovered SKAP forms a cognate complex with IQGAP1 in order to orchestrate directional cell migration, via coupling dynamic microtubule plus-ends, to the cell cortex^34^. Notably, SKAP functions in different cellular processes while interacting with various proteins. Given recent identification of single amino acid variation in SKAP^45^ and the involvement of SKAP variants in skin cancer development, it would be important to further elucidate the relationship among those variants at single molecule level^46^.

## Materials and Methods

### Cell Culture and Drug Treatments

HeLa and HEK293T cells, from the American Type Culture Collection (ATCC, Rockville, Maryland), were routinely maintained in advanced DMEM (Invitrogen) supplemented with 10% (vol/vol) FBS (Hyclone) and penicillin-streptomycin (100 IU/ml and 100 mg/ml, respectively, GIBCO). For cell synchronization, aliquots of HeLa cells were treated with 2 mM thymidine for 16 h, which allows cells to stay in G1/S. An alternative protocol is treating cells with 100 ng/ml nocodazole for 18 h to synchronize the cells in prometaphase. For cell treatment, MG132 (Sigma) was used at 20 μM for 2 h, while the GSK3β inhibitor SB415286 (Sigma) was used at 30 μM for 15 min. For *in vitro* kinase assay, SB415286 was used at 30 μM.

### Plasmids, RNAi and Transfection

The full-length SKAP mRNA was amplified as previously described^20^. GFP-tagged SKAP full-length and deletion truncations were cloned into pEGFP-C2 (Clontech), pmCherry-C2 (based on pEGFP-C2) and p3 × FLAG-myc-CMV™-24. Bacterial expression constructs of SKAP were cloned into pGEX-5X-3 (GE Healthcare). Human Kif2b complementary DNA (cDNA) was obtained from Duane A. Compton (Geisel School of Medicine at Dartmouth). Briefly, for the generation of GFP-tagged Kif2b, PCR-amplified cDNA was cloned into pEGFP-C2, and then subcloned into the pMAL-c2x vector (New England BioLabs). All Kif2b deletion mutants were generated by inserting corresponding PCR-amplified fragments into pEGFP-C2 and pMAL-c2x, respectively. Mutagenesis was performed using the site-directed mutagenesis kit (Vazyme Biotech Co., Ltd) according to the manufacturer’s instructions. All plasmids used were verified by sequencing (Invitrogen).

The siRNA sequence used for silencing SKAP is 5′-AGGCTACAAACCACTGAGTAA-3′ (L-022219–00; Thermo Fisher Scientific); Kif2b siRNA sequence is 5′-GGCAAGAAGAUUGACCUGG-3′, which was synthesized by GenePharma. As a control, a duplex targeting a scramble sequence was used^47^. The 21-mer oligonucleotide RNA duplexes were synthesized from Dharmacon Research, Inc. (Boulder, CO). All the plasmids and siRNAs were transfected into HeLa cells using Lipofectamine 2000 (Invitrogen) according to the user’s manual, and HEK293T cells were transfected with plasmids through the calcium phosphate method.

### Antibodies

The rabbit antibody against SKAP was generated using full-length recombinant proteins from bacteria according to a standard protocol as described previously^48^. Kif2c-related antibodies were described previously^40^. The following antibodies were obtained from commercial sources: Anti-Kif2b-rabbit antibody (Gift from Duane A. Compton); Anti-GSK3β mouse antibody (BD Biosciences); Anti-EB1 mouse antibody (BD Biosciences); Anti-FLAG mouse antibody (Sigma); Anti-GFP mouse antibody (BD Biosciences); Anti-His mouse antibody (Cell Signaling Technology); Anti-α-tubulin mouse antibody (DM1A, Sigma); Anti-Phosphoserine/threonine/tyrosine (SPM101) (Abcam). For all Western blots, signals were detected using HRP-conjugated anti-mouse or rabbit antibodies (Jackson ImmunoResearch).

### Recombinant Protein Expression

All recombinant proteins were expressed in *E. coli* stain Rosetta (DE3). Bacteria were grown in LB medium at 37 °C and protein expression was induced with 0.2 mM isopropyl β-D-1-thiogalactopyranoside (IPTG) for 16 h at 16 °C. Bacteria expressing MBP-Kif2b and its mutants were lysed in a MBP column buffer (20 mM Tris-HCl, pH 7.4; 200 mM NaCl, 1 mM EDTA) and incubated with amylose resin (NEB Biolabs) for 1.5 h at 4 °C. The resin was washed three times in the MBP column buffer and eluted with MBP column buffer supplemented with 10 mM maltose. Insect cells expressing 6 × His-GSK3β were lysed by sonication in 6 × His binding buffer (50 mM NaH_2_PO_4_, pH 8.0; 300 mM NaCl) containing 10 mM imidazole, and incubated with Ni-NTA agarose (Qiagen) for 1.5 h at 4 °C. The agarose was washed three times in 6 × His binding buffer supplemented with 20 mM imidazole and eluted with 6 × His binding buffer supplemented with 200 mM imidazole. Bacteria expressing GST-SKAP and its mutants were suspended and lysed by sonication in a phosphate buffered saline (PBS) buffer supplemented with 0.2% Triton X-100, and the resultant supernatant were incubated with glutathione-sepharose 4B (GE Healthcare Life Science) for 1.5 h at 4 °C. Then the resin was washed for three times. All the purification procedures were performed at 4 °C, and a protease inhibitor cocktail (Sigma) was added to prevent degradation of the proteins.

### *In Vitro* Pull-down Assay

GST fusion protein-bound sepharose beads were separately incubated for 2 h at 4 °C with human embryonic kidney (HEK) 293T cell lysates containing either ectopically-expressed GFP-tagged proteins, or their deletion mutants, purified MBP fusion proteins, or His-tagged fusion proteins expressed in bacterial cells on ice in PBS containing protease inhibitor cocktail (Sigma) and 0.1% Triton X-100. After the incubation, the beads were washed for three times with PBS containing 0.1% Triton X-100 and once more with PBS. Then the beads were boiled in SDS-PAGE sample buffer, the bound proteins were separated on SDS-PAGE.

### Immunoprecipitation

HEK293T cells were grown to ~70% confluency in DMEM and co-transfected with FLAG-SKAP and GFP-tagged proteins or their deletion mutants through the calcium phosphate transfection method. Cells were then harvested and solubilized by sonication on ice in a lysis buffer (50 mM HEPES, PH 7.4; 100 mM NaCl, 2 mM EGTA, 1 mM MgCl_2_. 1 mM DTT) containing 0.1% Triton X-100 and protease inhibitor cocktail (Sigma). The lysates were clarified by centrifugation at 13,000 rpm for 20 min at 4 °C. FLAG-SKAP was precipitated through incubation with the anti-FLAG antibody-agarose beads (Sigma). Beads were washed for three times with the lysis buffer containing 0.1% Triton X-100 and once with the lysis buffer. Finally, the beads were boiled and loaded onto 10% SDS-PAGE for Western blotting analysis with anti-FLAG and GFP antibodies, respectively.

HeLa cell lysates were prepared, clarified and incubated with SKAP rabbit IgG pre-bound Protein A/G-agarose as previously described^20^. After an extensive wash, Protein A/G-agarose were boiled in SDS-PAGE sample buffer, then a Western blotting analysis was performed to probe for corresponding proteins.

### *In vitro* kinase assay

GST and GST-tagged proteins were expressed in *E. coli*, purified by glutathione resin and eluted by 10 mM glutathione. The kinase reaction was performed in 30 μl 1× kinase buffer containing 0.1 μg GSK3β kinase (Invitrogen), 4 μg eluted GST protein or GST-fusion proteins, 0.5 μCi [γ-^32^P] ATP, and 50 μM ATP at 30 °C for 30 min. The reaction was stopped by SDS loading buffer and separated by SDS-PAGE. Then the gel was stained with Coomassie brilliant blue (CBB) and Western blot was performed. Phosphorylation was confirmed by an anti-p-ser/thr/tyr antibody.

To identify the GSK3β-mediated phosphorylation sites of SKAP, the kinase reaction was performed with ATP. Samples were removed and subjected to trypsin in-gel digest followed by collection of tryptic peptides for mass spectrometric analyses as described previously^49^.

### Immunofluorescence, Microscope, Image Processing and Data Analysis

HeLa cells were seeded onto sterile, acid-treated 12 mm coverslips on 24-well plates (Corning Glass Works), for transfection or drug treatment, and fixed with 3.7% formaldehyde in PBS for 10 min at 37 °C. For antibody staining, treated and fixed cells were permeabilized with 0.1% Triton-X100 in PBS for 5 min. Then the cells were washed with PBS and blocked with 1% BSA in PBS for 30 min at room temperature. Cells were subsequently incubated with indicated primary antibodies in a humidified chamber for 1 h at room temperature, then with secondary antibodies for 1 h at room temperature. DNA was stained with 4′,6-diamidino-2-phenylindole (DAPI, Sigma). Images were acquired using a DeltaVision wide-field deconvolution microscope (Applied Precision Inc., WA, USA) with a 60× objective lens, NA = 1.42, with optical sections acquired 0.2 μm apart in the Z-axis. Deconvoluted images from each focal plane were projected into a single image using SoftWorx software (Applied Precission). Images were taken at identical exposure time within each experiment and were acquired as 16-bit gray-scale images. After deconvolution, the images were exported as 24-bit RGB images and processed in Adobe Photoshop. Images shown in the same panel have been identically scaled. Statistical significance was determined by unpaired Student’s *t*-test.

### Live Cell Imaging

HeLa cells were cultured in a glass-bottomed culture dish (MatTek). During imaging, cells were maintained in CO_2_-independent media (Gibco) containing 10% (vol/vol) FBS and 1% (2 mM) glutamine in a sealed chamber at 37 °C. Images of living cells were taken with a DeltaVision RT system (Applied Precission). Images were prepared for publication using Adobe Photoshop. Measurements and statistical analyses were calculated using ImageJ software (NIH) and GraphPad Prism.

### MT depolymerization Assay

MTs were polymerized with 50 μM tubulin plus 1 mM GMPCPP (Jena Bioscience, Germany), as described previously^50^. Purified proteins were separately incubated with the reconstituted MTs in BRB80 containing 1 mM ATP and 1 mM DTT, for indicated time at 25 °C. The mixtures were then squashed onto coverslips and examined under a total internal reflection fluorescent (TIRF) microscope configured on an ELYRA system (Carl Zeiss).

## Additional Information

**How to cite this article**: Qin, B. *et al*. Phosphorylation of SKAP by GSK3β ensures chromosome segregation by a temporal inhibition of Kif2b activity. *Sci. Rep.* **6**, 38791; doi: 10.1038/srep38791 (2016).

**Publisher’s note:** Springer Nature remains neutral with regard to jurisdictional claims in published maps and institutional affiliations.

## Supplementary Material

Supplementary Information

## References

[b1] Lengauer C., Kinzler K. W. & Vogelstein B. Genetic instabilities in human cancers. Nature 396, 643–649, doi: 10.1038/25292 (1998). 9872311

[b2] Thompson S. L. & Compton D. A. Examining the link between chromosomal instability and aneuploidy in human cells. J Cell Biol 180, 665–672, doi: 10.1083/jcb.200712029 (2008). 18283116 PMC2265570

[b3] Thompson S. L. & Compton D. A. Proliferation of aneuploid human cells is limited by a p53-dependent mechanism. The Journal of cell biology 188, 369–381, doi: 10.1083/jcb.200905057 (2010). 20123995 PMC2819684

[b4] Bakhoum S. F., Thompson S. L., Manning A. L. & Compton D. A. Genome stability is ensured by temporal control of kinetochore-microtubule dynamics. Nat Cell Biol 11, 27–35, doi: 10.1038/ncb1809 (2009). 19060894 PMC2614462

[b5] Walker R. A. . Dynamic instability of individual microtubules analyzed by video light microscopy: rate constants and transition frequencies. The Journal of cell biology 107, 1437–1448 (1988). 3170635 10.1083/jcb.107.4.1437 PMC2115242

[b6] Maccioni R. B. & Cambiazo V. Role of microtubule-associated proteins in the control of microtubule assembly. Physiological reviews 75, 835–864 (1995). 7480164 10.1152/physrev.1995.75.4.835

[b7] Fukata Y. . CRMP-2 binds to tubulin heterodimers to promote microtubule assembly. Nature cell biology 4, 583–591, doi: 10.1038/ncb825 (2002). 12134159

[b8] Bunker J. M., Wilson L., Jordan M. A. & Feinstein S. C. Modulation of microtubule dynamics by tau in living cells: implications for development and neurodegeneration. Mol Biol Cell 15, 2720–2728, doi: 10.1091/mbc.E04-01-0062 (2004). 15020716 PMC420096

[b9] Cai S., Weaver L. N., Ems-McClung S. C. & Walczak C. E. Proper organization of microtubule minus ends is needed for midzone stability and cytokinesis. Current biology: CB 20, 880–885, doi: 10.1016/j.cub.2010.03.067 (2010). 20434340 PMC2869383

[b10] Ganem N. J. & Compton D. A. The KinI kinesin Kif2a is required for bipolar spindle assembly through a functional relationship with MCAK. J Cell Biol 166, 473–478, doi: 10.1083/jcb.200404012jcb.200404012 [pii] (2004). 15302853 PMC2172212

[b11] Ohi R., Burbank K., Liu Q. & Mitchison T. J. Nonredundant functions of Kinesin-13s during meiotic spindle assembly. Current biology: CB 17, 953–959, doi: 10.1016/j.cub.2007.04.057 (2007). 17509883

[b12] Sampath S. C. . The chromosomal passenger complex is required for chromatin-induced microtubule stabilization and spindle assembly. Cell 118, 187–202, doi: 10.1016/j.cell.2004.06.026 (2004). 15260989

[b13] Lan W. . Aurora B phosphorylates centromeric MCAK and regulates its localization and microtubule depolymerization activity. Current biology: CB 14, 273–286, doi: 10.1016/j.cub.2004.01.055 (2004). 14972678

[b14] Knowlton A. L., Lan W. & Stukenberg P. T. Aurora B is enriched at merotelic attachment sites, where it regulates MCAK. Current biology: CB 16, 1705–1710, doi: 10.1016/j.cub.2006.07.057 (2006). 16950107

[b15] Zhang L. . PLK1 phosphorylates mitotic centromere-associated kinesin and promotes its depolymerase activity. The Journal of biological chemistry 286, 3033–3046, doi: 10.1074/jbc.M110.165340 (2011). 21078677 PMC3024797

[b16] Maney T., Hunter A. W., Wagenbach M. & Wordeman L. Mitotic centromere-associated kinesin is important for anaphase chromosome segregation. The Journal of cell biology 142, 787–801 (1998). 9700166 10.1083/jcb.142.3.787 PMC2148171

[b17] Manning A. L. . The kinesin-13 proteins Kif2a, Kif2b, and Kif2c/MCAK have distinct roles during mitosis in human cells. Molecular biology of the cell 18, 2970–2979, doi: 10.1091/mbc.E07-02-0110 (2007). 17538014 PMC1949365

[b18] Kline-Smith S. L. & Walczak C. E. Mitotic spindle assembly and chromosome segregation: refocusing on microtubule dynamics. Molecular cell 15, 317–327, doi: 10.1016/j.molcel.2004.07.012 (2004). 15304213

[b19] Wang X. . Mitotic regulator SKAP forms a link between kinetochore core complex KMN and dynamic spindle microtubules. J Biol Chem 287, 39380–39390, doi: 10.1074/jbc.M112.406652 (2012). 23035123 PMC3501077

[b20] Huang Y. . CENP-E kinesin interacts with SKAP protein to orchestrate accurate chromosome segregation in mitosis. The Journal of biological chemistry 287, 1500–1509, doi: 10.1074/jbc.M111.277194 (2012). 22110139 PMC3256856

[b21] Fang L., Seki A. & Fang G. SKAP associates with kinetochores and promotes the metaphase-to-anaphase transition. Cell cycle 8, 2819–2827 (2009). 19667759 10.4161/cc.8.17.9514

[b22] Frame S. & Cohen P. GSK3 takes centre stage more than 20 years after its discovery. The Biochemical journal 359, 1–16 (2001). 11563964 10.1042/0264-6021:3590001 PMC1222116

[b23] Embi N., Rylatt D. B. & Cohen P. Glycogen synthase kinase-3 from rabbit skeletal muscle. Separation from cyclic-AMP-dependent protein kinase and phosphorylase kinase. European journal of biochemistry/FEBS 107, 519–527 (1980). 6249596

[b24] Cohen P. & Frame S. The renaissance of GSK3. Nature reviews. Molecular cell biology 2, 769–776, doi: 10.1038/35096075 (2001). 11584304

[b25] Jope R. S. & Johnson G. V. The glamour and gloom of glycogen synthase kinase-3. Trends in biochemical sciences 29, 95–102, doi: 10.1016/j.tibs.2003.12.004 (2004). 15102436

[b26] Jope R. S., Yuskaitis C. J. & Beurel E. Glycogen synthase kinase-3 (GSK3): inflammation, diseases, and therapeutics. Neurochemical research 32, 577–595, doi: 10.1007/s11064-006-9128-5 (2007). 16944320 PMC1970866

[b27] Woodgett J. R. Molecular cloning and expression of glycogen synthase kinase-3/factor A. The EMBO journal 9, 2431–2438 (1990). 2164470 10.1002/j.1460-2075.1990.tb07419.x PMC552268

[b28] Woodgett J. R. cDNA cloning and properties of glycogen synthase kinase-3. Methods in enzymology 200, 564–577 (1991). 1659660 10.1016/0076-6879(91)00172-s

[b29] Wakefield J. G., Stephens D. J. & Tavare J. M. A role for glycogen synthase kinase-3 in mitotic spindle dynamics and chromosome alignment. Journal of cell science 116, 637–646 (2003). 12538764 10.1242/jcs.00273

[b30] Tighe A., Ray-Sinha A., Staples O. D. & Taylor S. S. GSK-3 inhibitors induce chromosome instability. BMC cell biology 8, 34, doi: 10.1186/1471-2121-8-34 (2007). 17697341 PMC1976608

[b31] Lin C. C. . Characterization and functional aspects of human ninein isoforms that regulated by centrosomal targeting signals and evidence for docking sites to direct gamma-tubulin. Cell cycle 5, 2517–2527 (2006). 17102634 10.4161/cc.5.21.3404

[b32] Huang P., Senga T. & Hamaguchi M. A novel role of phospho-beta-catenin in microtubule regrowth at centrosome. Oncogene 26, 4357–4371, doi: 10.1038/sj.onc.1210217 (2007). 17260019

[b33] Xu W., Ge Y., Liu Z. & Gong R. Glycogen synthase kinase 3beta orchestrates microtubule remodeling in compensatory glomerular adaptation to podocyte depletion. The Journal of biological chemistry 290, 1348–1363, doi: 10.1074/jbc.M114.593830 (2015). 25468908 PMC4340382

[b34] Cao D. . Signaling Scaffold Protein IQGAP1 Interacts with Microtubule Plus-end Tracking Protein SKAP and Links Dynamic Microtubule Plus-end to Steer Cell Migration. The Journal of biological chemistry 290, 23766–23780, doi: 10.1074/jbc.M115.673517 (2015). 26242911 PMC4583049

[b35] Bastians H. Causes of Chromosomal Instability. Recent results in cancer research. Fortschritte der Krebsforschung. Progres dans les recherches sur le cancer 200, 95–113, doi: 10.1007/978-3-319-20291-4_5 (2015). 26376874

[b36] Akhmanova A. & Steinmetz M. O. Tracking the ends: a dynamic protein network controls the fate of microtubule tips. Nature reviews. Molecular cell biology 9, 309–322, doi: 10.1038/nrm2369 (2008). 18322465

[b37] Gadde S. & Heald R. Mechanisms and molecules of the mitotic spindle. Current biology: CB 14, R797–805, doi: 10.1016/j.cub.2004.09.021 (2004). 15380094

[b38] Chretien D., Fuller S. D. & Karsenti E. Structure of growing microtubule ends: two-dimensional sheets close into tubes at variable rates. The Journal of cell biology 129, 1311–1328 (1995). 7775577 10.1083/jcb.129.5.1311 PMC2120473

[b39] Mandelkow E. M., Mandelkow E. & Milligan R. A. Microtubule dynamics and microtubule caps: a time-resolved cryo-electron microscopy study. The Journal of cell biology 114, 977–991 (1991). 1874792 10.1083/jcb.114.5.977 PMC2289108

[b40] Jiang K. . TIP150 interacts with and targets MCAK at the microtubule plus ends. EMBO reports 10, 857–865, doi: 10.1038/embor.2009.94 (2009). 19543227 PMC2699393

[b41] Cooper J. R., Wagenbach M., Asbury C. L. & Wordeman L. Catalysis of the microtubule on-rate is the major parameter regulating the depolymerase activity of MCAK. Nature structural & molecular biology 17, 77–82, doi: 10.1038/nsmb.1728 (2010). PMC2909650 19966798

[b42] Ems-McClung S. C. . Aurora B inhibits MCAK activity through a phosphoconformational switch that reduces microtubule association. Curr Biol 23, 2491–2499, doi: 10.1016/j.cub.2013.10.054S0960-9822(13)01329-8 [pii] (2013). 24291095 PMC3897551

[b43] Xia P. . Aurora A orchestrates entosis by regulating a dynamic MCAK-TIP150 interaction. Journal of molecular cell biology 6, 240–254, doi: 10.1093/jmcb/mju016 (2014). 24847103 PMC4034728

[b44] Tamura N. . A proteomic study of mitotic phase-specific interactors of EB1 reveals a role for SXIP-mediated protein interactions in anaphase onset. Biology open 4, 155–169, doi: 10.1242/bio.201410413 (2015). 25596275 PMC4365484

[b45] Su Z. D. . De novo identification and quantification of single amino-acid variants in human brain. Journal of molecular cell biology 6, 421–433, doi: 10.1093/jmcb/mju031 (2014). 25007923

[b46] Jin J., Lian T., Sunney Xie X. & Su X. D. High-accuracy mapping of protein binding stability on nucleosomal DNA using a single-molecule method. Journal of molecular cell biology 6, 438–440, doi: 10.1093/jmcb/mju033 (2014). 25035518

[b47] Lou Y. . NEK2A interacts with MAD1 and possibly functions as a novel integrator of the spindle checkpoint signaling. The Journal of biological chemistry 279, 20049–20057, doi: 10.1074/jbc.M314205200 (2004). 14978040

[b48] Wang H. . Human Zwint-1 specifies localization of Zeste White 10 to kinetochores and is essential for mitotic checkpoint signaling. The Journal of biological chemistry 279, 54590–54598, doi: 10.1074/jbc.M407588200 (2004). 15485811

[b49] Han G. . Comprehensive and reliable phosphorylation site mapping of individual phosphoproteins by combination of multiple stage mass spectrometric analysis with a target-decoy database search. Analytical chemistry 81, 5794–5805, doi: 10.1021/ac900702g (2009). 19522514

[b50] Shao H. . Spatiotemporal dynamics of Aurora B-PLK1-MCAK signaling axis orchestrates kinetochore bi-orientation and faithful chromosome segregation. Scientific reports 5, 12204, doi: 10.1038/srep12204 (2015). 26206521 PMC4513279

