# Supplementary material for: Phosphorylation of SKAP by GSK3β ensures chromosome segregation by a temporal inhibition of Kif2b activity
Source: Sci Rep. 2016 Dec 16;6:38791. doi: 10.1038/srep38791 (PMC5159797; doi:10.1038/srep38791)
Supplement: Supplementary Information [file srep38791-s1.pdf]

Supplementary Information

Phosphorylation of SKAP by GSK3β ensures chromosome segregation by a temporal inhibition of Kif2b activity

Bo Qin[*1], Dan Cao[*1], Huihui Wu[1], Fei Mo[1], Hengyi Shao[1], Jan Chu[3], Michael Powell[3], Felix Aikhionbare[3], Dongmei Wang[1,2], Chuanhai Fu[1,2], Ping He[4], Weijun Pan[5], Wenwen Wang[1,2], Xing Liu[1,2], & Xuebiao Yao[1,2]

[1]Anhui Key Laboratory of Cellular Dynamics and Chemical Biology, University of Science & Technology of China, Hefei 230027, China; [2]Center of Excellence on Molecular Cell Sciences, Chinese Academy of Sciences, Hefei 230026, China; [3]Molecular Imaging Center, Atlanta Clinical & Translational Science Institute, Atlanta, GA 30310; [4]Guangzhou Women and Children's Medical Center, Guangzhou 510623, China; [5]Shanghai Institutes for Biological Sciences, Chinese Academy of Sciences, Shanghai 200031, China

*These authors equally contributed to this work.

Correspondence and requests: xing1017@ustc.edu.cn; wwwang@mail.ustc.edu.cn

Tel: +86-551-63606304; Fax: +86-551-63607141

Supplemental Figure S1

ATPase rates were determined by measuring the liberated phosphate over time in reactions containing 1 mM ATP and 0.3 μM Kif2b. EnzChek Phosphate Assay Kit (Invitrogen) was used to quantify phosphate release by measuring absorbance at 360 nm. Purified SKAP and its mutants were detected by SDS-PAGE. FLAG-Kif2b was obtained from 293T cells.

Supplemental Figure S2

Bacterially recombinant GST-SKAP, GST and the positive control GST-myc were incubated with GSK3β kinase in an in vitro phosphorylation reaction as described in “Materials and Methods”. Samples were separated by SDS-PAGE and stained with Coomassie Brilliant Blue to ensure equal amounts of GST-SKAP and GST used in the reactions. The gels were then dried and exposed to an X-ray film.

Supplemental Figure S3

Full-length blots/gels of Figure 1b, 3a, 3b, 4a, 4f, 6c and 7a

Supplemental Figure S1

a

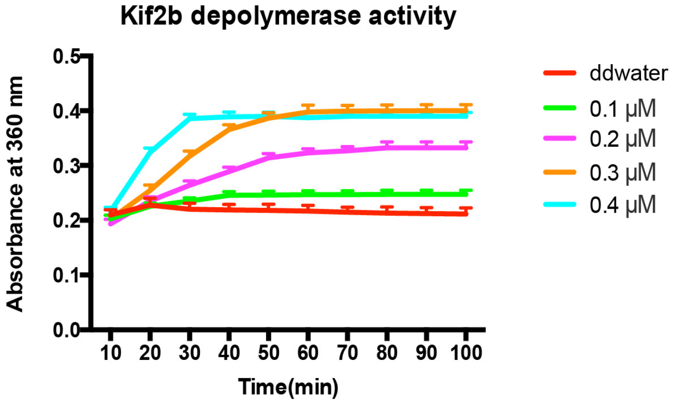

b

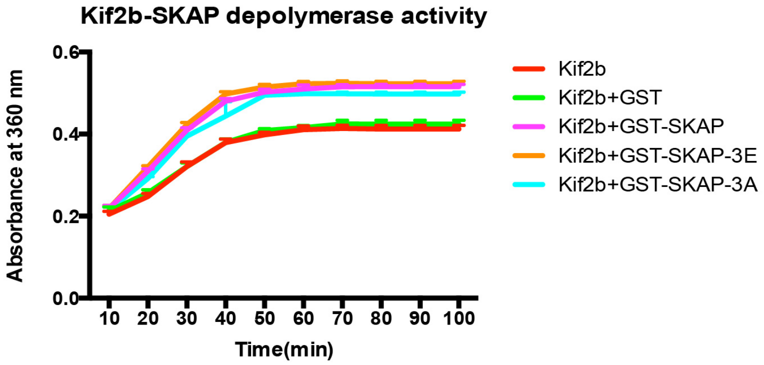

Supplemental Figure S2

a

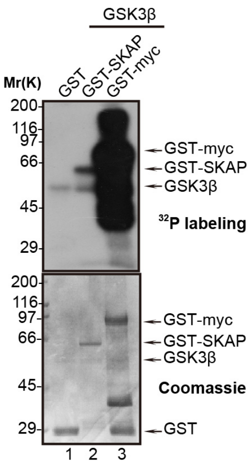

Supplemental Figure S3

Fig 1b

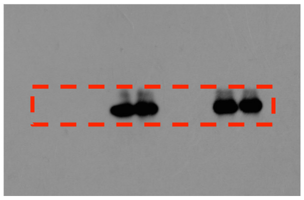

Flag Blot

Fig 3b

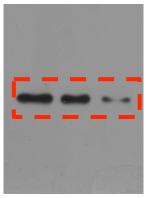

SKAP Blot

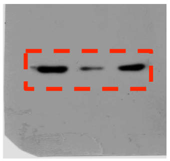

Kif2b Blot

Fig 4a

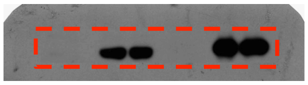

Flag Blot

Fig 4f

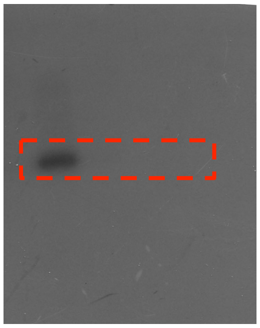

^{32}P labeling

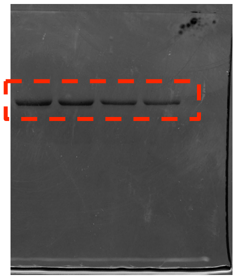

CBB staining

Fig 3a

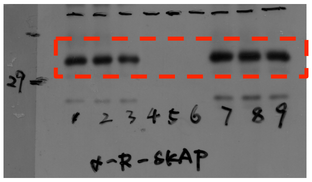

SKAP Blot

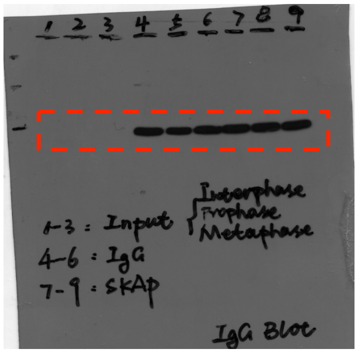

IgG Blot

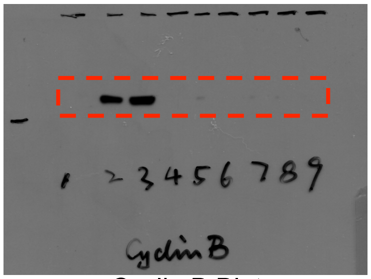

Cyclin B Blot

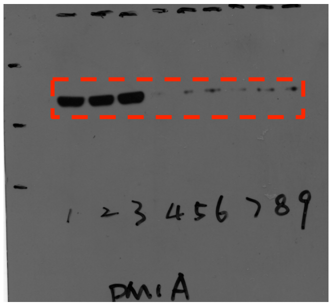

Tubulin Blot

Fig 6c

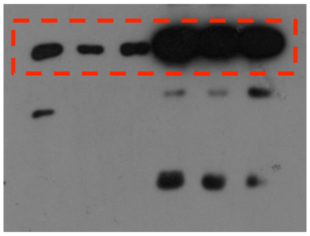

Flag Blot

Fig 7a

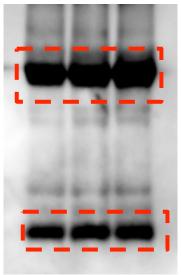

SKAP Blot
